# The Value of FujiLAM in the Diagnosis of Tuberculosis: A Systematic Review and Meta-Analysis

**DOI:** 10.3389/fpubh.2021.757133

**Published:** 2021-11-25

**Authors:** Zhenzhen Li, Xiang Tong, Sitong Liu, Jirong Yue, Hong Fan

**Affiliations:** ^1^Health Management Center, West China Hospital/West China School of Medicine, Sichuan University, Chengdu, China; ^2^Department of Respiratory Medicine and Critical Care Medicine, West China Hospital/West China School of Medicine, Sichuan University, Chengdu, China; ^3^Department of Geriatrics and National Clinical Research Center for Geriatrics, West China Hospital/West China School of Medicine, Sichuan University, Chengdu, China

**Keywords:** tuberculosis, FujiLAM, sensitivity, specificity, meta-analysis

## Abstract

**Background:** Timely and accurate diagnosis of tuberculosis (TB) remains a major challenge. Lipoarabinomannan (LAM) is a specific component of the cell envelope of *Mycobacterium tuberculosis* and is also a potential biomarker for the diagnosis of TB. Recently, the Fujifilm SILVAMP TB LAM test (FujiLAM), as a novel urine lateral flow LAM test, was developed for the diagnosis of TB and is convenient and timely. Because of a difference in the diagnostic value of FujiLAM in the original studies, we conducted a meta-analysis to comprehensively assess the diagnostic value of FujiLAM in TB.

**Data Sources:** We performed a literature search using the PubMed and EMBASE databases and commercial Internet search engines to identify studies.

**Methods:** Searches of databases using relevant terms (“tuberculosis” or “TB”) and (“Fujifilm SILVAMP TB LAM” or “FujiLAM”) were performed. Screening, study reviewing, data extracting and assessing data quality was performed independently by two reviewers. We calculated the pooled sensitivity, specificity, positive likelihood ratio, negative likelihood ratio, and diagnostic odds ratio. To minimize potential heterogeneity, we performed subgroup analyses.

**Results:** Nine articles were included in the meta-analysis. When using the microbiological reference standard (MRS), the results showed that the sensitivity and specificity of FujiLAM were 0.70 and 0.93, respectively, in adults with TB, while the sensitivity and specificity of FujiLAM in children with TB were 0.51 and 0.87. When using a comprehensive reference standard (CRS), the sensitivity and specificity of FujiLAM in adults with TB were 0.59 and 0.96, respectively, while the results showed that the sensitivity and specificity of FujiLAM in children with TB were 0.27 and 0.86, respectively. Subgroup analysis showed that FujiLAM had higher diagnostic sensitivity in patients with human immunodeficiency virus infection or CD4 cell counts < 200 cells/μL, both in adults and children.

**Conclusions:** This meta-analysis suggests that FujiLAM has a high value in the diagnosis of adults with TB.

## Introduction

Tuberculosis (TB) is caused by *Mycobacterium tuberculosis (M. tuberculosis)*, which is still the leading cause of death from a single source of infection in the world and is also the leading cause of death for people living with human immunodeficiency virus (HIV) (1). In recent years, the emergence of multidrug-resistant and extensively drug-resistant *M. tuberculosis* has been a major threat to global TB control (1, 2). Rapid diagnosis and appropriate treatment are important to reduce TB transmission, mortality, and drug resistance (3). There are several methods of TB diagnosis, including smear and culture, radiological examination, and molecular tests (4–6). These methods have their own advantages and disadvantages; in particular, etiological culturing is time-consuming, and the test results are often related to whether high-quality sputum samples can be obtained (5).

Lipoarabinomannan (LAM) is a specific component of the cell envelope of *M. tuberculosis* (7). During *M. tuberculosis* infection, LAM exists in a variety of body fluids; therefore, it can be an ideal candidate biomarker for detecting *M. tuberculosis* (8). More meaningfully, Nathavitharana et al. found that using lateral flow lipoarabinomannan assay (LF-LAM) as part of a TB diagnostic testing strategy likely reduced mortality and probably resulted in a slight increase in anti-tuberculosis therapy initiation in HIV patients (9). In recent years, a novel detection technology, the Fujifilm SILVAMP TB LAM test (FujiLAM; Fujifilm, Tokyo, Japan) has been developed (10). This method is similar to the urine-based point-of-care test, Alere Determine TB LAM Ag (AlereLAM; Abbott, Palatine, IL, USA), which is based on the detection of lipoarabinomannan antigen in urine (10). However, FujiLAM uses silver-amplified immunochromatography on the lateral flow strip, and its analytical sensitivity is 30 times higher than that of AlereLAM (11). More importantly, FujiLAM only requires a simple 5-step process to obtain results, and the time required to acquire the test results is < 1 h (12). In the past 3 years, a series of studies on FujiLAM for TB diagnosis have been published, but the results of the different studies have not been consistent (13, 14). Therefore, we conducted a meta-analysis to investigate the utility of FujiLAM in the diagnosis of TB. In our study, we have included all the published original studies, comprehensively assessed the diagnostic value of FujiLAM in TB, and we performed different subgroup analyses of possible influencing factors.

## Methods

### Literature Search

We conducted systematic literature searches using the PubMed and EMBASE databases and several commercial Internet search engines, such as Google Scholar and Baidu Scholar, to determine the value of FujiLAM in the diagnosis of TB. The most recent searches performed on May 1, 2021 used the following terms: (“tuberculosis” or “TB”) and (“Fujifilm SILVAMP TB LAM” or “FujiLAM”). Results were limited to papers published in English or Chinese. In addition, the authors screened the references of the identified articles.

### Study Selection

The inclusion criteria were as follows: (1) studies of the diagnostic value of FujiLAM for TB, designed as a diagnostic accuracy study; (2) studies conducted in humans; (3) studies that provide sufficient data (true positives/negatives, false positives/negatives) to count pooled sensitivity and specificity; and (4) if there was duplication of data, only the most complete study was included. The exclusion criteria were as follows: (1) the study did not provide available data for counting effect size or was missing other essential information; and (2) review, abstract, unpublished data or overlapping study. All analyses in this meta-analysis were based on previously published studies; therefore, ethical approval or patient consent was not required.

### Data Extraction

Two independent authors (Zhenzhen Li and Xiang Tong) used a pre-designed Microsoft® Excel® table to extract detailed information and data from each study. If there was any inconsistency, the third author (Sitong Liu) further reviewed these articles and a final consensus was reached through discussion. The following data were extracted from the identified studies: the name of the first author; year of publication; age and sex distribution of participants; sample size of patients and control groups; TB type; proportion of patients with HIV; sensitivity and specificity with their 95% confidence intervals (CI); and the number of true positives, false positives, false negatives, and true negatives in each study. All data were extracted according to two groups: microbiological reference standard (MRS) and comprehensive reference standard (CRS).

### Definitions

In the MRS group, participants classified as having confirmed TB were considered as reference standard positive, while participants without TB and with possible TB were considered negative (13). In the CRS group, participants with possible TB were reclassified as positive, together with confirmed TB (15).

### Quality Evaluation

The quality of the included studies was assessed using the Quality Assessment of Diagnostic Accuracy Studies-2 (QUADAS-2) conducted by two authors (Zhenzhen Li and Xiang Tong). The scale for assessing quality is based on four key points: patient selection, index test, reference standard, and flow and timing in the primary study. Each item was assessed based on the risk of bias, and the first three items were assessed based on concerns regarding applicability. Any differences were settled by consensus. The quality assessment was performed using the software RevMan version 5.3.

### Statistical Method

All data were analyzed using Meta-Disc software 1.4 version. As in previous studies (16), the random effects model was used to pool data from individual studies to obtain the following test accuracy measurements: sensitivity, specificity, positive likelihood ratio, negative likelihood ratio, and diagnostic odds ratio. Summary receiver operator characteristic (SROC) curves summarized the sensitivity and specificity of each study to depict the overall diagnostic accuracy of FujiLAM, while the area under the curve was obtained from the SROC chart. The between-study heterogeneity was investigated using the chi-squared (χ^2^)-based *Q*-test and *I*^2^ statistics test. If *P* < 0.10 or *I*^2^> 50%, significant heterogeneity was suggested. If there was significant heterogeneity, subgroup analysis was performed according to the most likely influencing factors (such as HIV status, CD4 cell levels and sample types).

## Results

### General Characteristics

**Figure 1:**
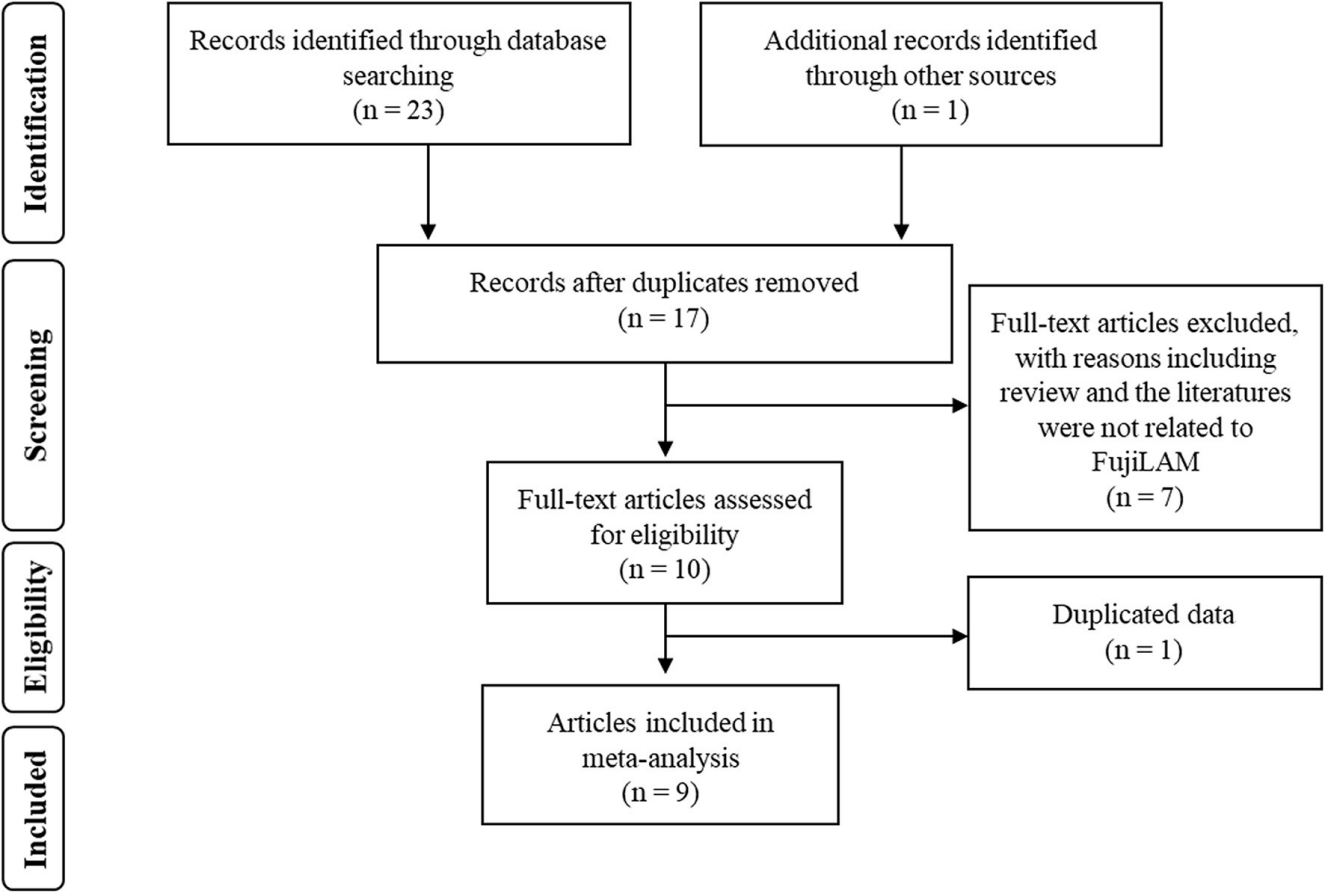
The flow diagram of included and excluded studies.

**Figure 2:**
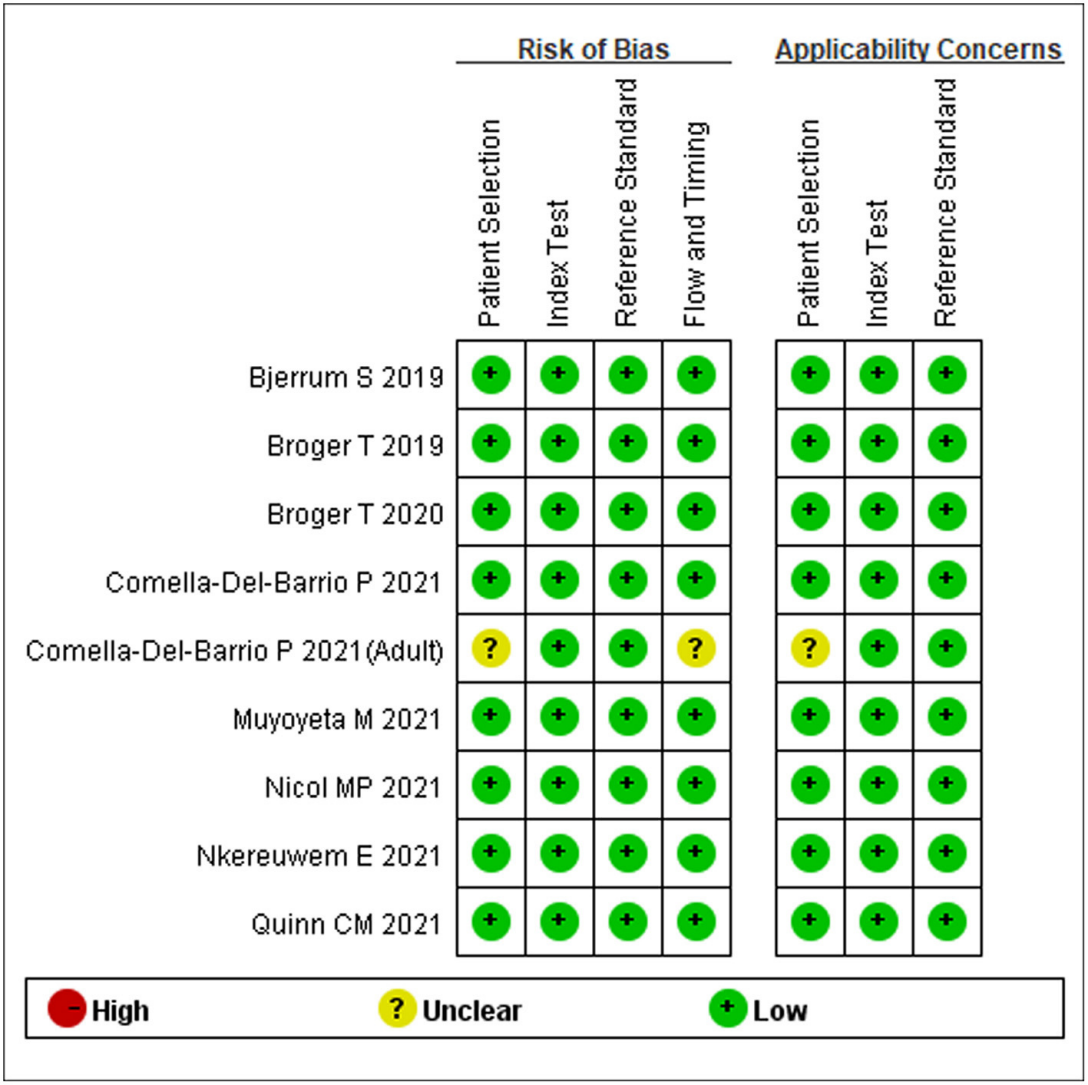
Summary of quality assessment of included studies by the QUADAS-2 assessment.

We initially identified 24 studies. Seven studies were excluded because they were duplicate findings in different databases. After full-text screening of the remaining studies, five were excluded because they were reviews, two were excluded because they did not evaluate the diagnostic value of FujiLAM, and one article was excluded because there were potentially repeat data (Figure 1). Finally, a total of nine articles that described the value of FujiLAM in the diagnosis of TB patients were identified (11–14, 17–21). Among them, three studies reported the diagnostic value of FujiLAM in pediatric patients with TB, while the rest were the diagnostic value of FujiLAM in adult patients with TB. Additionally, the patients included in one study all had extra pulmonary TB (EPTB), and the patients included in other studies were mainly diagnosed with pulmonary TB (PTB). All studies were conducted in Africa, a region with a high TB burden. The remaining features are summarized in Supplementary Table 1. Among the included studies, according to the quality evaluation using QUADAS-2, eight studies had low risk, and one had unclear risk (Figure 2).

### Overall Diagnostic Accuracy

In the MRS group, a total of eight studies analyzed the diagnostic value of FujiLAM in TB. The results showed that the pooled sensitivity and specificity were 0.66 (95% CI: 0.58–0.74, *I*^2^ = 76.1%) and 0.92 (95% CI: 0.88–0.96, *I*^2^= 88.3%), respectively. In the CRS group, 7 studies analyzed the diagnostic value of FujiLAM in TB, and the results showed that the pooled sensitivity and specificity were 0.42 (95% CI: 0.32–0.57, *I*^2^= 94.3%) and 0.95 (95% CI: 0.92–0.99, *I*^2^= 72.5%), respectively. There was significant heterogeneity in the overall analysis; therefore, we conducted a subgroup analysis of the diagnostic value of FujiLAM by likely influencing factors in adults and children with TB.

### Diagnostic Value of FujiLAM in Adult Patients With TB (MRS)

**Figure 3:**
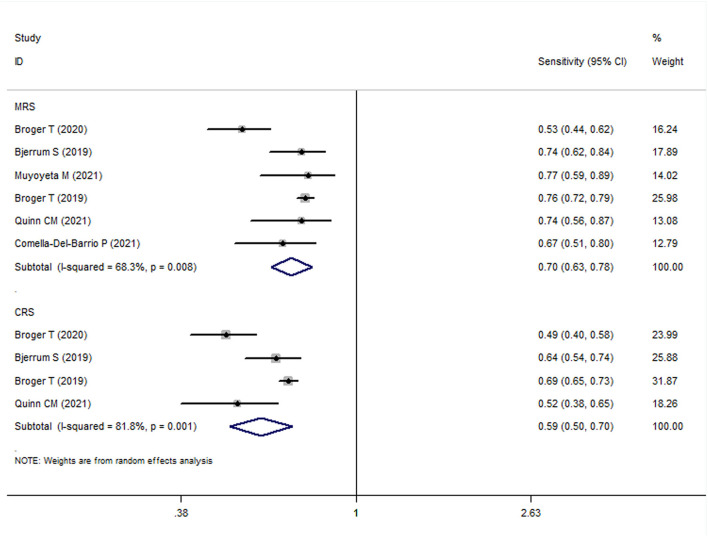
The meta-analysis results of the sensitivity of FujiLAM in the diagnosis of adult patients with tuberculosis.

**Figure 4:**
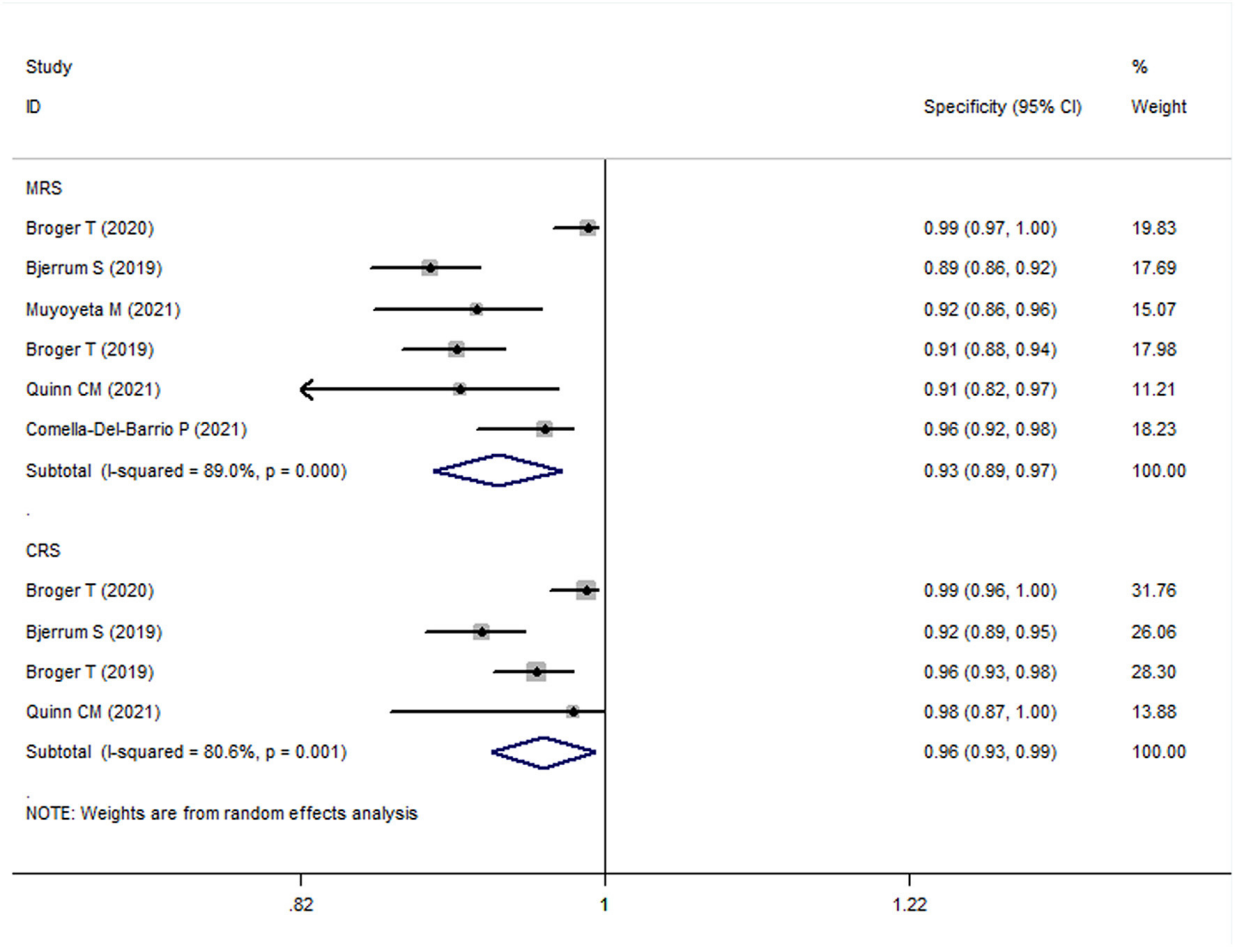
The meta-analysis results of the specificity of FujiLAM in the diagnosis of adult patients with tuberculosis.

The meta-analysis results showed that the sensitivity and specificity of FujiLAM in adults with TB were 0.70 (95% CI: 0.63–0.78, *I*^2^ = 68.3%) and 0.93 (95% CI: 0.88–0.98, *I*^2^ = 89.0%), respectively (Figures 3, 4). Subgroup analysis showed that the sensitivity and specificity of FujiLAM for patients with CD4 cell counts >200 cells/μL were 0.46 (95% CI: 0.36–0.56, *I*^2^ = 0%) and 0.98 (95% CI: 0.96–0.99, *I*^2^ = 0%), respectively. For patients with CD4 cell counts < 200 cells/μL, the sensitivity and specificity of FujiLAM were 0.81 (95% CI: 0.77–0.84, *I*^2^ = 0%) and 0.84 (95% CI: 0.80–0.88, *I*^2^ = 54.5%), respectively. Additionally, the results showed that the sensitivity and specificity of FujiLAM were in patients with HIV infection (0.75, 95% CI: 0.72–0.79, *I*^2^ = 0%; and 0.90, 95% CI: 0.88–0.92, *I*^2^ = 0%, respectively), and were in patients without HIV infection (0.58, 95% CI: 0.51–0.66, *I*^2^ = 55.0%; 0.98, 95% CI: 0.97–0.99, *I*^2^ = 40.5%, respectively). Using frozen urine samples and fresh urine samples for FujiLAM test, it was found that the sensitivity was 0.73 (95% CI: 0.68–0.78, *I*^2^ = 32.6%) and 0.64 (95% CI: 0.44–0.92, *I*^2^ = 86.2%), and the specificity was 0.92 (95% CI: 0.88–0.96, *I*^2^ = 81.4%) and 0.96 (95% CI: 0.89–1.0, *I*^2^ = 83.9%), respectively. One study used cerebrospinal fluid samples for FujiLAM test, and the sensitivity and specificity were 0.74 (95% CI: 0.59–0.92, *I*^2^ = 0%) and 0.91 (95% CI: 0.84–0.99, *I*^2^ = 0%), respectively. In the sputum smear microscopy (SSM)-positive group, the sensitivity of FujiLAM were 0.82 (95% CI: 0.66–1.02, *I*^2^ = 80.5%), while the sensitivity of FujiLAM were 0.45 (95% CI: 0.26–0.80, *I*^2^ = 81.7%) in SSM-negative group. The results of the meta-analysis are summarized in Table 1.

**Table 1:** Pooled results of FujiLAM in the diagnosis of adult patients with tuberculosis.

	**Sensitivity**	**Specificity**	**Positive LR**	**Negative LR**	**Diagnostic OR**	**SROC**
**MRS**						
Overall	0.70 (0.63–0.78)	0.93 (0.89–0.97)	/	/	/	/
HIV infected	0.75 (0.72–0.79)	0.90 (0.88–0.92)	7.75 (6.29–9.55)	0.22 (0.24–0.31)	29.25 (21.36–40.04)	0.88
HIV uninfected	0.58 (0.51–0.66)	0.98 (0.97–0.99)	31.07 (12.57–76.79)	0.40 (0.29–0.55)	92.51 (39.10–218.92)	0.93
CD4 cell > 200	0.46 (0.36–0.56)	0.98 (0.96–0.99)	21.7 (10.04–46.92)	0.56 (0.46–0.67)	38.73 (16.0–93.71)	/
CD4 cell ≤ 200	0.81 (0.77–0.84)	0.84 (0.80–0.88)	5.1 (3.41–7.63)	0.22 (0.19–0.27)	24.31 (15.89–37.17)	/
Frozen urine samples	0.73 (0.68–0.78)	0.92 (0.88–0.96)	/	/	/	/
Fresh urine samples	0.64 (0.44–0.92)	0.96 (0.89–1.00)	/	/	/	/
Cerebrospinal fluid	0.74 (0.59–0.92)	0.91 (0.84–0.99)	/	/	/	/
SSM positive	0.82 (0.66–1.0)	/	/	/	/	/
SSM negative	0.45 (0.26–0.80)	/	/	/	/	/
**CRS**						
Overall	0.59 (0.50–0.70)	0.96 (0.93–0.99)	/	/	/	/
HIV infected	0.67 (0.64–0.70)	0.94 (0.92–0.96)	12.74 (5.83–27.87)	0.39 (0.30–0.50)	34.95 (16.04–76.17)	0.83
CD4 cell > 200	0.36 (0.28–0.45)	0.99 (0.97–1.0)	27.52 (10.0–75.68)	0.65 (0.57–0.74)	42.91 (14.51–126.95)	/
CD4 cell ≤ 200	0.75 (0.71–0.78)	0.90 (0.86–0.93)	7.71 (2.29–25.96)	0.29 (0.21–0.41)	25.64 (6.79–96.75)	/
Frozen urine samples	0.69 (0.65–0.72)	0.94 (0.91–0.97)	/	/	/	/
Fresh urine samples	0.49 (0.41–0.59)	0.99 (0.97–1.00)	/	/	/	/
Cerebrospinal fluid	0.52 (0.40–0.68)	0.98 (0.91–1.00)	/	/	/	/

*LR, likelihood ratio; OR, odds ratio; SROC, summary receiver operator characteristic curve; MRS, microbiological reference standard; CRS, comprehensive reference standard; HIV, human immunodeficiency virus; SSM, sputum smear microscopy*.

### Diagnostic Value of FujiLAM in Adult Patients With TB (CRS)

The meta-analysis results showed that the sensitivity and specificity of FujiLAM in adult TB were 0.59 (95% CI: 0.50–0.70, *I*^2^ = 81.8%) and 0.96 (95% CI: 0.93–0.99, *I*^2^ = 80.6%), respectively (Figures 3, 4). Subgroup analysis showed that the sensitivity and specificity of FujiLAM for patients with CD4 cell counts >200 cells/μL were 0.36 (95% CI: 0.28–0.45, *I*^2^ = 0%) and 0.99 (95% CI: 0.97–1.0, *I*^2^ = 0%), respectively. For patients with CD4 cell counts < 200 cells/μL, the sensitivity and specificity of FujiLAM were 0.75 (95% CI: 0.71–0.78, *I*^2^ = 13.4%) and 0.90 (95% CI: 0.86–0.93, *I*^2^ = 85.4%), respectively. In HIV-infected patients, the results showed that the sensitivity and specificity of FujiLAM were 0.67 (95% CI: 0.64–0.70, *I*^2^ = 72.9%) and 0.94 (95% CI: 0.92–0.96, *I*^2^ = 61.3%). Using frozen urine samples and fresh urine samples for FujiLAM test, it was found that the sensitivity was 0.69 (95% CI: 0.65–0.72, *I*^2^ = 0%) and 0.49 (95% CI: 0.41–0.59, *I*^2^ = 0%), and the specificity was 0.94 (95% CI: 0.91–0.97, *I*^2^ = 65.9%), and 0.99 (95% CI: 0.97–1.0, *I*^2^ = 0%), respectively. One study used cerebrospinal fluid samples for FujiLAM test, and the sensitivity and specificity were 0.52 (95% CI: 0.40–0.68 *I*^2^ = 0%) and 0.98 (95% CI: 0.91–1.0, *I*^2^ = 0%), respectively. The results of the meta-analysis are summarized in Table 1.

### Diagnostic Value of FujiLAM in Pediatric Patients With TB

**Figure 5:**
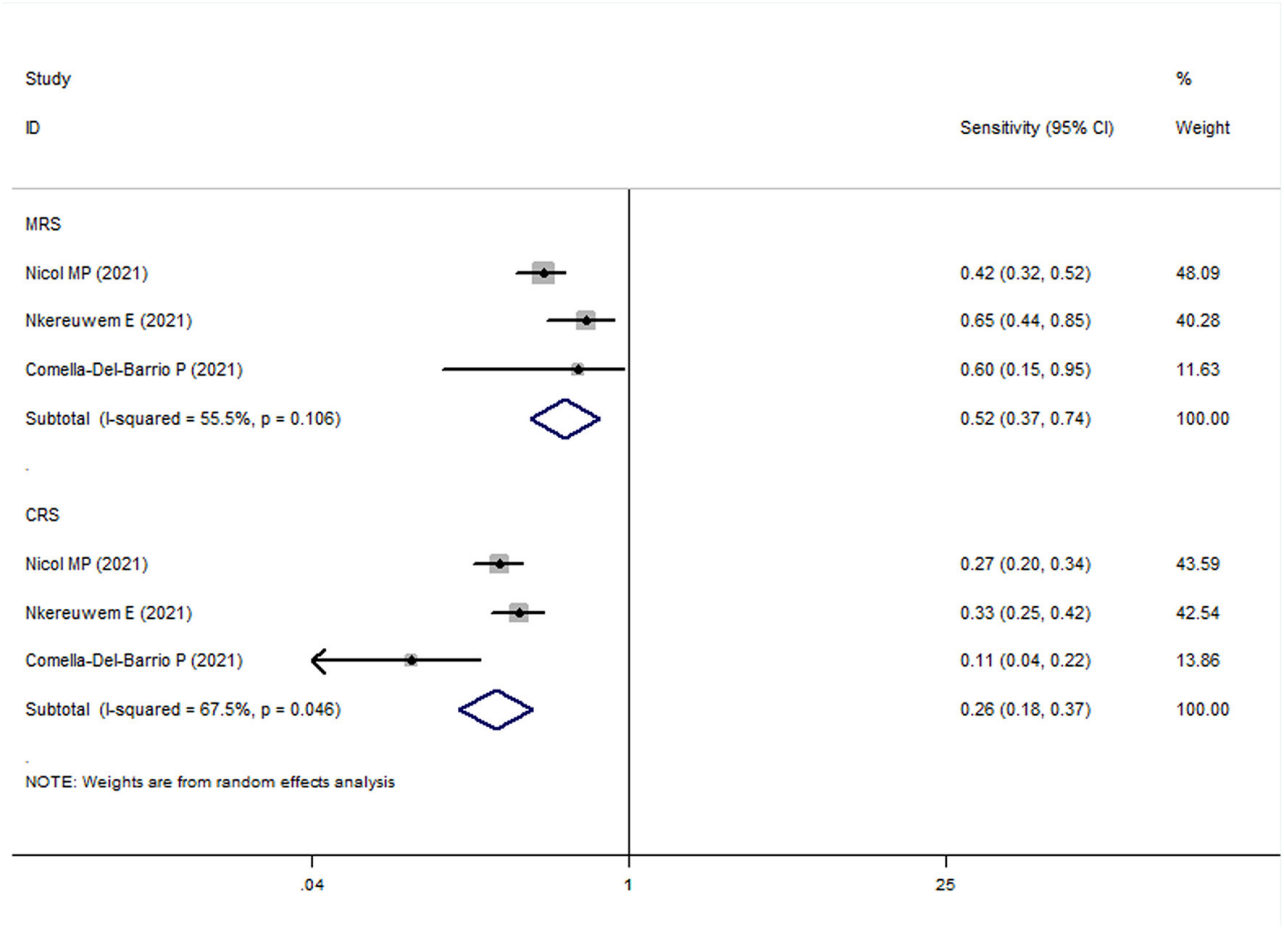
The meta-analysis results of the sensitivity of FujiLAM in the diagnosis of pediatric patients with tuberculosis.

**Figure 6:**
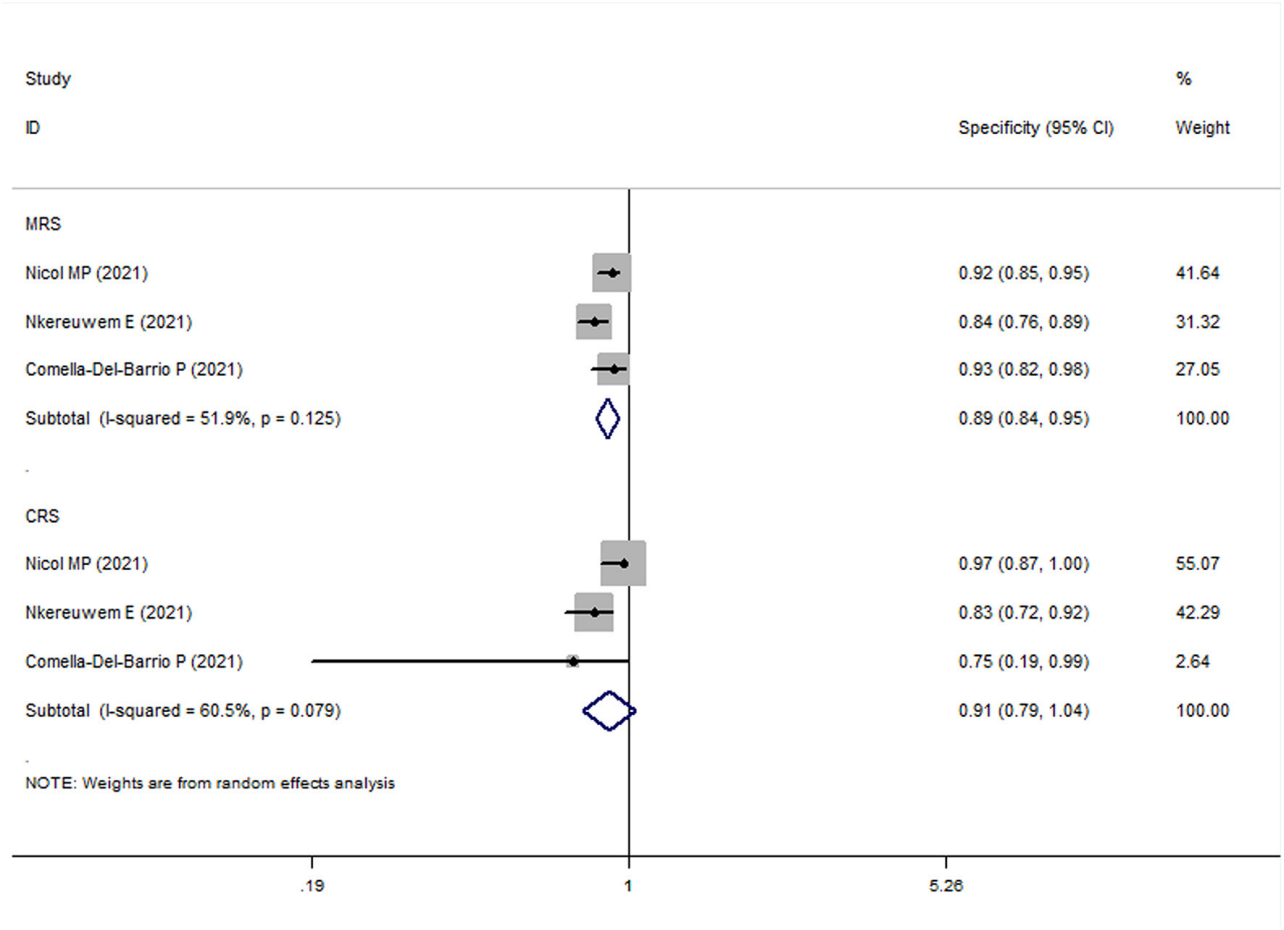
The meta-analysis results of the specificity of FujiLAM in the diagnosis of pediatric patients with tuberculosis.

In all pediatric studies, FujiLAM tests were performed with frozen urine samples. For MRS group, the overall results showed that the sensitivity and specificity of FujiLAM in children with TB were 0.51 (95% CI: 0.43–0.59, *I*^2^ = 71.8%) and 0.87 (95% CI: 0.84–0.90, *I*^2^ = 68.5%), respectively (Figures 5, 6). The subgroup analysis results showed that the sensitivity and specificity of FujiLAM in patients with HIV infection were 0.58 (95% CI: 0.41–0.73 and *I*^2^ = 0%) and 0.80 (95% CI: 0.68–0.89 and *I*^2^ = 60.6%), respectively, and in patients without HIV infection were 0.54 (95% CI: 0.44–0.64 and *I*^2^ = 71.0%), and 0.88 (95% CI: 0.85–0.91 and *I*^2^ = 33.2%), respectively (Table 2).

**Table 2:** Pooled results of FujiLAM in the diagnosis of pediatric patients with tuberculosis.

	**Sensitivity**	**Specificity**	**Positive LR**	**Negative LR**	**Diagnostic OR**	**SROC**
**MRS**						
Overall	0.51 (0.43–0.59)	0.87 (0.84–0.90)	4.37 (3.33–5.72)	0.53 (0.37–0.75)	9.14 (5.78–14.44)	0.83
HIV infected	0.58 (0.41–0.73)	0.80 (0.68–0.89)	3.63 (0.77–17.03)	0.50 (0.35–0.73)	6.93 (1.27–37.84)	/
HIV uninfected	0.54 (0.44–0.64)	0.88 (0.85–0.91)	5.13 (3.79–6.96)	0.50 (0.31–0.80)	11.03 (6.48–18.77)	0.89
**CRS**						
Overall	0.27 (0.23–0.32)	0.86 (0.82–0.90)	2.09 (0.53–8.22)	0.78 (0.71–0.87)	2.68 (0.59–12.25)	0.45
HIV uninfected	0.27 (0.20–0.34)	0.87 (0.82–0.91)	1.34 (0.25–7.14)	0.87 (0.59–1.28)	1.54 (0.19–12.13)	

*LR, likelihood ratio; OR, odds ratio; SROC, summary receiver operator characteristic curve; MRS, microbiological reference standard; CRS, comprehensive reference standard; HIV, human immunodeficiency virus*.

For the CRS group, the overall results showed that the sensitivity and specificity of FujiLAM in children with TB were 0.27 (95% CI: 0.23–0.32, *I*^2^ = 82.9%) and 0.86 (95% CI: 0.82–0.90, *I*^2^ = 70.8%), respectively. The subgroup analysis results showed that the sensitivity and specificity of FujiLAM in patients without HIV infection were 0.27 (95% CI: 0.20–0.34, *I*^2^ = 91.0%) and 0.87 (95% CI: 0.82–0.91, *I*^2^ = 0%), respectively (Table 2).

## Discussion

TB remains a severe public health threat to humans. Because of the inherent characteristics of *M. tuberculosis*, the diagnosis of TB by etiology is still difficult (5). As a novel method, FujiLAM is based on the detection of antigens of *M. tuberculosis* in urine, which is convenient, fast, and has attracted much attention in recent years (12, 14). We conducted a meta-analysis to assess the diagnostic value of FujiLAM for TB. The main results of the current meta-analysis are as follows. First, the overall results show that FujiLAM has moderate sensitivity for the diagnosis of TB, but the specificity is high. The high specificity suggests that FujiLAM may play a role as a rule-in test for TB. Second, subgroup analysis of multiple factors was carried out in this study, the results showed that the sensitivity of FujiLAM for TB diagnosis was moderate to high in different subgroups. Finally, the sensitivity and specificity of FujiLAM in the diagnosis of adults with TB was higher than that of children with TB. However, it should be noted that because the number of included studies is still small, the conclusions should be interpreted with caution.

In our meta-analysis, compared with that in the CRS group, the sensitivity of FujiLAM in the MRS group was higher; the specificity was similar between the MRS and CRS groups. We speculate that patients with positive pathogens may be more prone to having LAM antigen in urine, and FujiLAM detects LAM antigen in urine, which may be the reason for the higher diagnostic sensitivity of FujiLAM in the MRS group. However, due to the lack of sufficient research on FujiLAM, further validation is needed.

In the present meta-analysis, we analyzed the diagnostic value of FujiLAM in children and adults with TB. It was found that the sensitivity of FujiLAM in the diagnosis of childhood TB was low to moderate, while the specificity was still high, but the specificity was also lower than that of adults with TB. As Nkereuwem et al. mentioned, this may be because of the higher amount of expected contamination from perineal flora in the process of urine collection in children than in adults (11). It is worth mentioning that because of its high specificity and because it is very convenient and operable to collect urine for the detection of *M. tuberculosis*, FujiLAM is a suitable method for the diagnosis of TB in children, especially when it is difficult to obtain high-quality sputum samples.

We also performed subgroup analyses based on HIV infection status and CD4 count levels. The results showed that the sensitivity of FujiLAM was higher in patients with HIV infection and CD4 cell counts < 200 cells/μL, regardless of MRS or CRS grouping. This may be associated with the extremely low immune function in patients with HIV infection or CD4 cell counts < 200 cells/μL, so that the load of *M. tuberculosis* is higher or patients are more prone to disseminated TB. Muyoyeta et al. found that in HIV-positive patients, FujiLAM detected TB in all patients with low, medium, or high sputum Xpert-positive results, but missed TB in three patients with a very low Xpert-positive result, indicating an association between FujiLAM and the *M. tuberculosis* burden in HIV-positive patients (20). In HIV-negative patients, there was no significant correlation between FujiLAM and Xpert Ultra semi-quantitative results (20). In addition, we compared the diagnostic value of FujiLAM in sputum-positive and sputum-negative patients. The meta-analysis showed that FujiLAM had higher sensitivity in sputum-positive patients, which further indicates that it might be more likely to detect *M. tuberculosis* in the urine of patients with a positive etiology. These raw data are only from a few studies, so they need to be further verified in the future.

In fact, Broger et al. conducted a meta-analysis showing that FujiLAM identified a substantially higher proportion of TB patients in people living with HIV than LF-LAM in 2020 (22). The advantages of our study were that we comprehensively evaluated the diagnostic value of FujiLAM in all tuberculosis patients through meta-analysis, and we also conducted subgroup analysis for possible influencing factors, including HIV status, CD4 cell levels and sample types. In addition, we further separately evaluated the diagnostic value of FujiLAM in SSM-positive and negative patients. However, this meta-analysis has some limitations. First, there are few primary studies on FujiLAM, all of which come from Africa, which may lead to some bias, and the results may apply only to the African population. Second, the diagnosis of TB may be influenced by many other factors, including age, education level, and medical compliance. Because of the limited data provided by the primary studies, it is difficult to perform further subgroup analyses of confounding factors. Third, although most studies included patients with PTB, one study included patients with EPTB, which may have led to bias in the overall results. Although we conducted a subgroup analysis in this study, the heterogeneity of the results was still significant, suggesting that there may be great differences among primary studies, and more high-quality studies are needed to verify the results in the future.

In conclusion, this meta-analysis suggests that FujiLAM has a high value in the diagnosis of adults with TB, especially in patients with HIV infection or CD4 cell count < 200 cells/μL. Although the diagnostic value of FujiLAM in children with TB is lower than that in adults, FujiLAM is very convenient as it uses urine testing; therefore, this will help to promote TB diagnosis in children. Further large-scale real-world studies are needed to confirm these results in the future.

## Data Availability Statement

The original contributions presented in the study are included in the article/Supplementary Material, further inquiries can be directed to the corresponding authors.

## Author Contributions

JY and HF designed the study, coordinated the study, and directed its implementation. ZL, XT, and SL collected data and conducted the follow-up work. ZL and XT wrote the manuscript. All authors have read and approved the final manuscript.

## Funding

This study was supported by National Key R&D Program of China (2017YFC1309703), a project funded by the China Postdoctoral Science Foundation (2020M673259), and the Post-Doctor Research Project, West China Hospital, Sichuan University (2020HXBH013).

## Conflict of Interest

The authors declare that the research was conducted in the absence of any commercial or financial relationships that could be construed as a potential conflict of interest.

## Publisher's Note

All claims expressed in this article are solely those of the authors and do not necessarily represent those of their affiliated organizations, or those of the publisher, the editors and the reviewers. Any product that may be evaluated in this article, or claim that may be made by its manufacturer, is not guaranteed or endorsed by the publisher.
