# Supplementary material for: The Value of FujiLAM in the Diagnosis of Tuberculosis: A Systematic Review and Meta-Analysis
Source: Front Public Health. 2021 Nov 25;9:757133. doi: 10.3389/fpubh.2021.757133 (PMC8655683; doi:10.3389/fpubh.2021.757133)
Supplement: Supplementary file 1 [file Table_1.DOCX]

Supplementary table 1 The study characteristics of FujiLAM in the Diagnosis of Tuberculosis

| Author | Year | Type |  | Sample size | No. of patient with HIV | Age | Confirmed TB | Unconfirmed TB | Unlikely TB | Models | Sensitivity | LCI | UCI | Specificity | LCI | UCI | ALL | TP | FP | FN | TN |
| --- | --- | --- | --- | --- | --- | --- | --- | --- | --- | --- | --- | --- | --- | --- | --- | --- | --- | --- | --- | --- | --- |
| Nicol MP | 2021 | children | PTB | 204 | 40 | 3.75 (1.75-7.42) | 84 | 81 | 39 | MRS | 0.417 | 0.317 | 0.523 | 0.917 | 0.853 | 0.954 | MRS | 35 | 10 | 49 | 110 |
|  |  |  |  |  |  |  |  |  |  | CRS | 0.267 | 0.205 | 0.339 | 0.974 | 0.868 | 0.995 | CRS | 44 | 1 | 121 | 38 |
|  |  |  |  |  |  |  |  |  |  |  |  |  |  |  |  |  | No-HIV |  |  |  |  |
|  |  |  |  |  |  |  |  |  |  |  |  |  |  |  |  |  | MRS | 26 | 10 | 41 | 110 |
|  |  |  |  |  |  |  |  |  |  |  |  |  |  |  |  |  | CRS | 35 | 1 | 113 | 38 |
|  |  |  |  |  |  |  |  |  |  |  |  |  |  |  |  |  | CD4 counts |  |  |  |  |
|  |  |  |  |  |  |  |  |  |  |  |  |  |  |  |  |  | ＞200 | 11 | 0 | 9 | 11 |
|  |  |  |  |  |  |  |  |  |  |  |  |  |  |  |  |  | ＜200 | 4 | 1 | 1 | 3 |
|  |  |  |  |  |  |  |  |  |  |  |  |  |  |  |  |  | HIV (MRS) |  |  |  |  |
|  |  |  |  |  |  |  |  |  |  |  |  |  |  |  |  |  | Infection | 15 | 1 | 10 | 14 |
|  |  |  |  |  |  |  |  |  |  |  |  |  |  |  |  |  | Uninfection | 20 | 9 | 29 | 96 |
| Nkereuwem E | 2021 | children | PTB | 415 | 61 | 5.6 (2.3-9.3) | 63 | 113 | 239 | MRS | 0.649 | 0.437 | 0.852 | 0.838 | 0.765 | 0.894 | MRS | 40 | 55 | 23 | 297 |
|  |  |  |  |  |  |  |  |  |  | CRS | 0.329 | 0.246 | 0.419 | 0.833 | 0.718 | 0.917 | CRS | 58 | 37 | 118 | 202 |
|  |  |  |  |  |  |  |  |  |  |  |  |  |  |  |  |  | HIV (MRS) |  |  |  |  |
|  |  |  |  |  |  |  |  |  |  |  |  |  |  |  |  |  | Infection | 8 | 11 | 7 | 35 |
|  |  |  |  |  |  |  |  |  |  |  |  |  |  |  |  |  | Uninfection | 31 | 40 | 15 | 258 |
|  |  |  |  |  |  |  |  |  |  |  |  |  |  |  |  |  | HIV (CRS) |  |  |  |  |
|  |  |  |  |  |  |  |  |  |  |  |  |  |  |  |  |  | Infection | 14 | 5 | 30 | 12 |
|  |  |  |  |  |  |  |  |  |  |  |  |  |  |  |  |  | Uninfection | 43 | 28 | 86 | 187 |
| Broger T | 2020 | adult | PTB | 372 | 0 | 32 (25–47) Y | 111 | 10 | 251 | MRS | 0.532 | 0.439 | 0.622 | 0.989 | 0.967 | 0.996 | MRS | 59 | 3 | 52 | 258 |
|  |  |  |  |  |  |  |  |  |  | CRS | 0.488 | 0.4 | 0.576 | 0.988 | 0.965 | 0.996 | CRS | 59 | 3 | 62 | 248 |
|  |  |  |  |  |  |  |  |  |  | SSM (MRS) | |  |  |  |  |  |  |  |  |  |  |
|  |  |  |  |  |  |  |  |  |  | Negative | 0.2 | 0.1 | 0.359 |  |  |  |  |  |  |  |  |
|  |  |  |  |  |  |  |  |  |  | Positive | 0.684 | 0.573 | 0.778 |  |  |  |  |  |  |  |  |
| Bjerrum S | 2019 | adult | TB | 450 | 450 | 38 (31–45) | 66 | 32 | 352 | MRS | 0.742 | 0.62 | 0.842 | 0.893 | 0.858 | 0.922 | MRS | 49 | 41 | 17 | 343 |
|  |  |  |  |  |  |  |  |  |  | CRS | 0.643 | 0.54 | 0.737 | 0.923 | 0.89 | 0.949 | CRS | 63 | 27 | 35 | 325 |
|  |  |  |  |  |  |  |  |  |  |  |  |  |  |  |  |  | CD4 counts (MRS) | |  |  |  |
|  |  |  |  |  |  |  |  |  |  |  |  |  |  |  |  |  | ＞200 | 8 | 3 | 7 | 181 |
|  |  |  |  |  |  |  |  |  |  |  |  |  |  |  |  |  | ＜200 | 39 | 35 | 10 | 153 |
|  |  |  |  |  |  |  |  |  |  |  |  |  |  |  |  |  | CD4 counts (CRS) | |  |  |  |
|  |  |  |  |  |  |  |  |  |  |  |  |  |  |  |  |  | ＞200 | 9 | 2 | 13 | 175 |
|  |  |  |  |  |  |  |  |  |  |  |  |  |  |  |  |  | ＜200 | 50 | 24 | 22 | 141 |
| Muyoyeta M | 2021 | adult | TB | 151 | 68 | 37 (28-43) | 34 | NA | NA | MRS | 0.77 | 0.59 | 0.89 | 0.92 | 0.86 | 0.96 | MRS | 26 | 9 | 8 | 108 |
|  |  |  |  |  |  |  |  |  |  |  |  |  |  |  |  |  | HIV (MRS) |  |  |  |  |
|  |  |  |  |  |  |  |  |  |  |  | SSM (MRS) | |  |  |  |  | Infection | 9 | 6 | 3 | 50 |
|  |  |  |  |  |  |  |  |  |  |  | Negative | 0.68 | 0.43 | 0.87 |  |  | Uninfection | 15 | 3 | 5 | 58 |
|  |  |  |  |  |  |  |  |  |  |  | Positive | 0.87 | 0.6 | 0.98 |  |  |  |  |  |  |  |
| Comella-Del-Barrio P | 2021 | children | TB | 59 | 0 | 6.33 (4.83–10.08) | 5 | 50 | 4 | MRS | 0.6 | 0.15 | 0.95 | 0.93 | 0.82 | 0.98 | MRS | 3 | 4 | 2 | 50 |
|  |  |  |  |  |  |  |  |  |  | CRS | 0.11 | 0.04 | 0.22 | 0.75 | 0.19 | 0.99 | CRS | 6 | 1 | 49 | 3 |
| Broger T | 2019 | adult | TB | 968 | 968 | 35 (30–42) | 600 | 91 | 277 | MRS | 0.758 | 0.722 | 0.792 | 0.908 | 0.876 | 0.937 | MRS | 455 | 33 | 145 | 335 |
|  |  |  |  |  |  |  |  |  |  | CRS | 0.69 | 0.654 | 0.725 | 0.957 | 0.93 | 0.98 | CRS | 477 | 11 | 214 | 266 |
|  |  |  |  |  |  |  |  |  |  |  |  |  |  |  |  |  | CD4 counts (MRS) | |  |  |  |
|  |  |  |  |  |  |  |  |  |  |  |  |  |  |  |  |  | ＞200 | 37 | 4 | 46 | 144 |
|  |  |  |  |  |  |  |  |  |  |  |  |  |  |  |  |  | ＜200 | 415 | 29 | 98 | 190 |
|  |  |  |  |  |  |  |  |  |  |  |  |  |  |  |  |  |  |  |  |  |  |
| Quinn CM | 2021 | adult | EPTB | 101 | 101 | 33 (26–40) | 34 | 19 | 48 | MRS | 0.74 | 0.56 | 0.87 | 0.91 | 0.82 | 0.97 | MRS | 25 | 6 | 9 | 61 |
|  |  |  |  |  |  |  |  |  |  | CRS | 0.52 | 0.38 | 0.65 | 0.98 | 0.87 | 1 | CRS | 30 | 1 | 28 | 42 |
| Comella-Del-Barrio P | 2021 | adult | TB | 204 | 70 | 37.0±12.8 | 45 | NA | 159 | MRS | 0.667 | 0.51 | 0.8 | 0.962 | 0.92 | 0.98 | MRS | 30 | 6 | 15 | 153 |
|  |  |  |  |  |  |  |  |  |  |  |  |  |  |  |  |  | HIV (MRS) |  |  |  |  |
|  |  |  |  |  |  |  |  |  |  |  |  |  |  |  |  |  | Infection | 7 | 4 | 3 | 56 |
|  |  |  |  |  |  |  |  |  |  |  |  |  |  |  |  |  | Uninfection | 23 | 1 | 12 | 97 |
|  |  |  |  |  |  |  |  |  |  |  |  |  |  |  |  |  | CD4 counts (MRS) |  |  |  |  |
|  |  |  |  |  |  |  |  |  |  |  |  |  |  |  |  |  | ＞200 | 2 | 1 | 10 | 2 |
|  |  |  |  |  |  |  |  |  |  |  |  |  |  |  |  |  | ＜200 | 30 | 3 | 5 | 1 |
